# Atypical soft palate pyogenic granuloma in an elderly anticoagulated patient presenting with acute oropharyngeal hemorrhage

**DOI:** 10.1093/jscr/rjag044

**Published:** 2026-02-05

**Authors:** Ammir Abuzahra, Ruba Atawneh, Raya Abu Ayyash, Jana Dwaik, Siwar Abu Sakout, Jehad Kashkeesh

**Affiliations:** Department of Otolaryngology – Head and Neck Surgery, Faculty of Medicine, Hebron University, Hebron P7060796, Palestine; Department of Otolaryngology – Head and Neck Surgery, Faculty of Medicine, Hebron University, Hebron P7060796, Palestine; Department of Otolaryngology – Head and Neck Surgery, Faculty of Medicine, Hebron University, Hebron P7060796, Palestine; Department of Otolaryngology – Head and Neck Surgery, Faculty of Medicine, Hebron University, Hebron P7060796, Palestine; Department of Otolaryngology – Head and Neck Surgery, Faculty of Medicine, Hebron University, Hebron P7060796, Palestine; Department of Otolaryngology – Head and Neck Surgery, Halhul Governmental Hospital, Hebron P7401216, Palestine

**Keywords:** pyogenic granuloma, soft palate, anticoagulation, oropharyngeal hemorrhage, elderly patient

## Abstract

Pyogenic granuloma (PG) is a benign lobular capillary proliferation of mucocutaneous tissues that usually arises in response to local irritation. It typically occurs on the gingiva, with extragingival sites such as the soft palate being very rare. We report a 69-year-old female on Apixaban who presented with sudden, profuse oropharyngeal bleeding. Examination revealed a friable, sessile erythematous mass on the right soft palate that was actively bleeding. The lesion was surgically excised with electrocautery under local anesthesia after appropriate management of anticoagulation. Histopathology confirmed lobular capillary hemangioma (PG). No recurrence was observed at follow-up. This case highlights the importance of prompt recognition and complete excision of PG in anticoagulated patients, as even small lesions may precipitate life threating hemorrhage.

## Introduction

Pyogenic granuloma (PG), also known as lobular capillary hemangioma, is a common benign vascular lesion of skin and mucous membranes [1]. It is characterized by rapid growth of granulation tissue with abundant capillaries, often arising after local irritation or minor trauma [2]. The term ‘PG’ is a misnomer, as the lesion is neither purulent nor a true granuloma histologically [1]. In the oral cavity, PG most frequently develops on the gingiva (about 75% of cases) [3]. Lip, tongue, and mucosal sites are less common, and involvement of the soft palate is particularly rare [4]. PG lesions are typically red, friable, and prone to bleeding on minor provocation [5]. They may occur at any age but are more common in young adults and are more common in females, possibly due to hormonal influences [2]. Treatment is surgical excision with removal of the lesion base and cauterization to minimize recurrence [5]. We report an unusual case of soft palate PG in an elderly patient on anticoagulation, presenting as an acute bleeding oropharyngeal emergency.

## Case report

**Figure 1:**
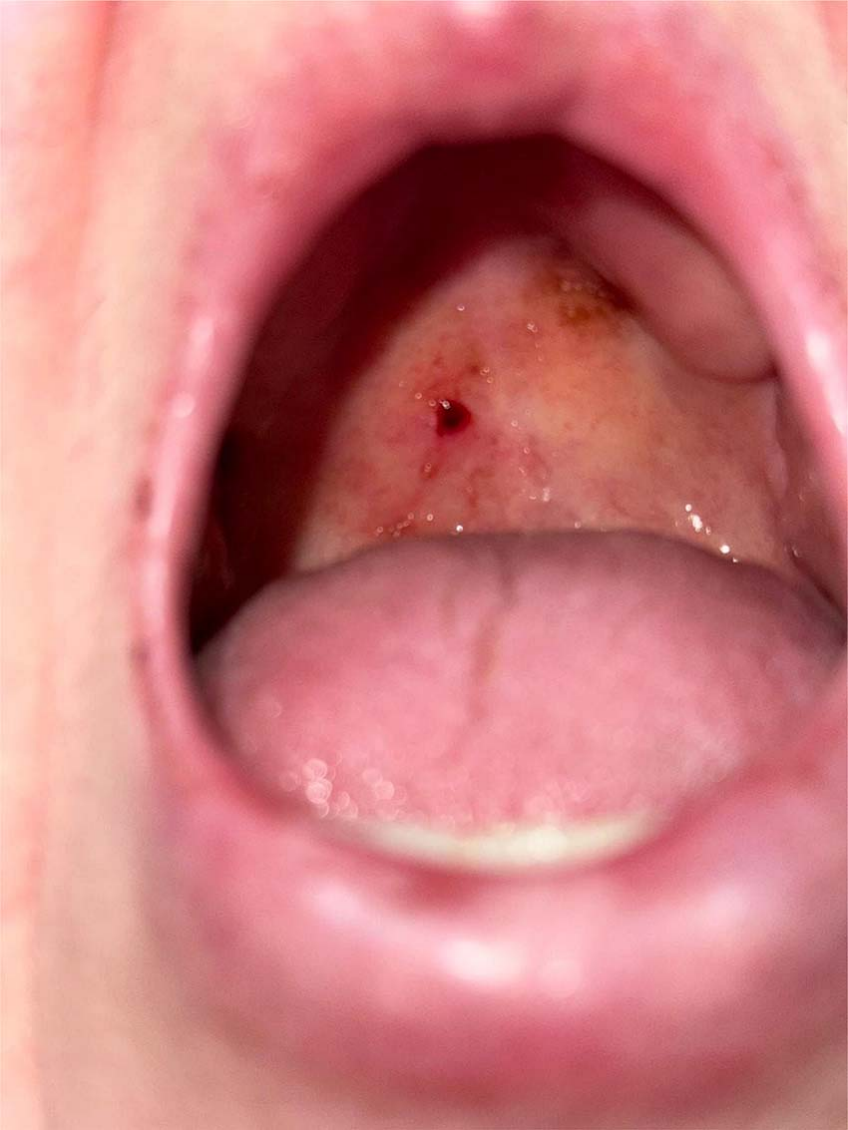
Clinical image of the oropharynx showing a sessile erythematous lesion on the right soft palate. The lesion appears as a friable, lobulated mass arising directly from the palatal mucosa without a pedicle.

**Figure 2:**
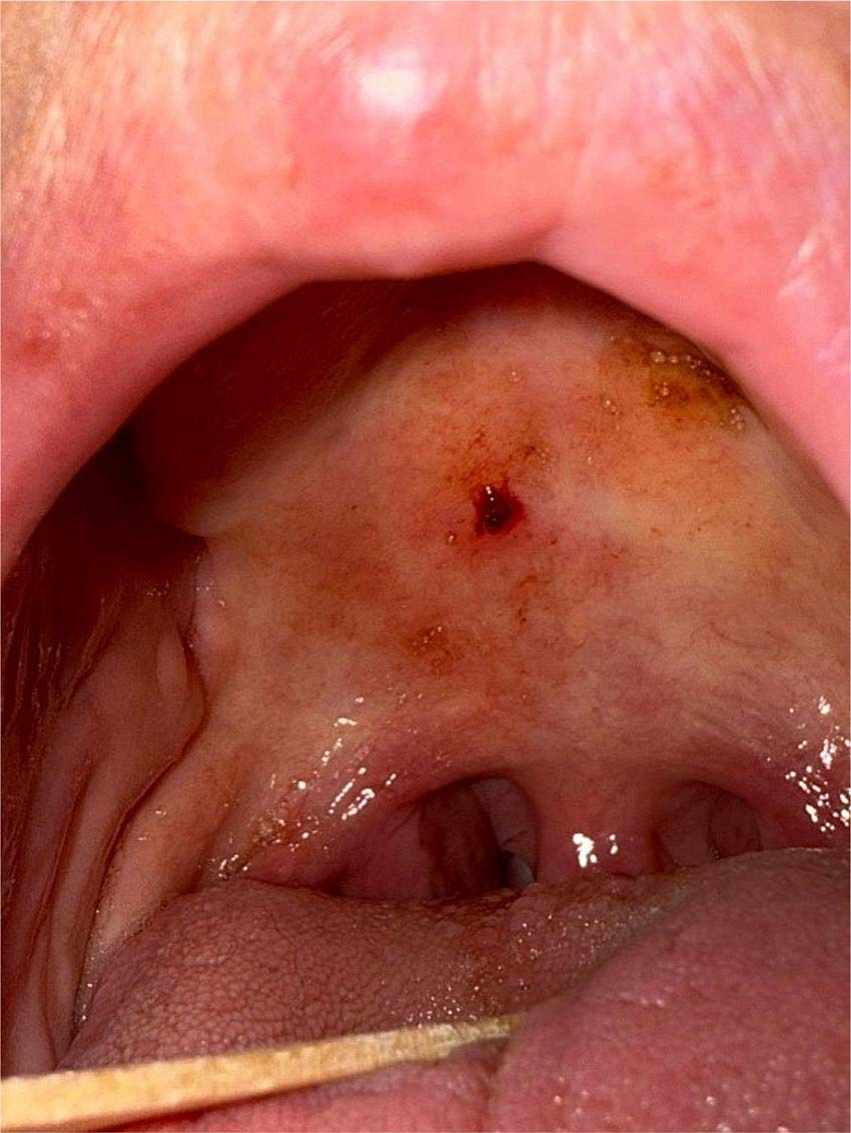
Closer view of the lesion with a tongue depressor for retraction. The lobulated, vascular surface of the PG is evident.

A 69-year-old woman with atrial fibrillation on long-term apixaban therapy presented to the emergency department with the sudden onset of heavy bleeding from the mouth and throat. She reported light-headedness and had noticed a mass at the back of her throat. On examination, she appeared pale and anxious. Her blood pressure was 90/60 mmHg and heart rate 110/min. Oropharyngeal examination revealed a bright-red sessile mass measuring approximately 0.5 cm on the right lateral soft palate, which bled on contact (Fig. 1). The lesion was friable with a lobulated surface, and a closer inspection demonstrated its highly vascular, irregular appearance (Fig. 2). No additional oral or oropharyngeal abnormalities were identified.

**Figure 3:**
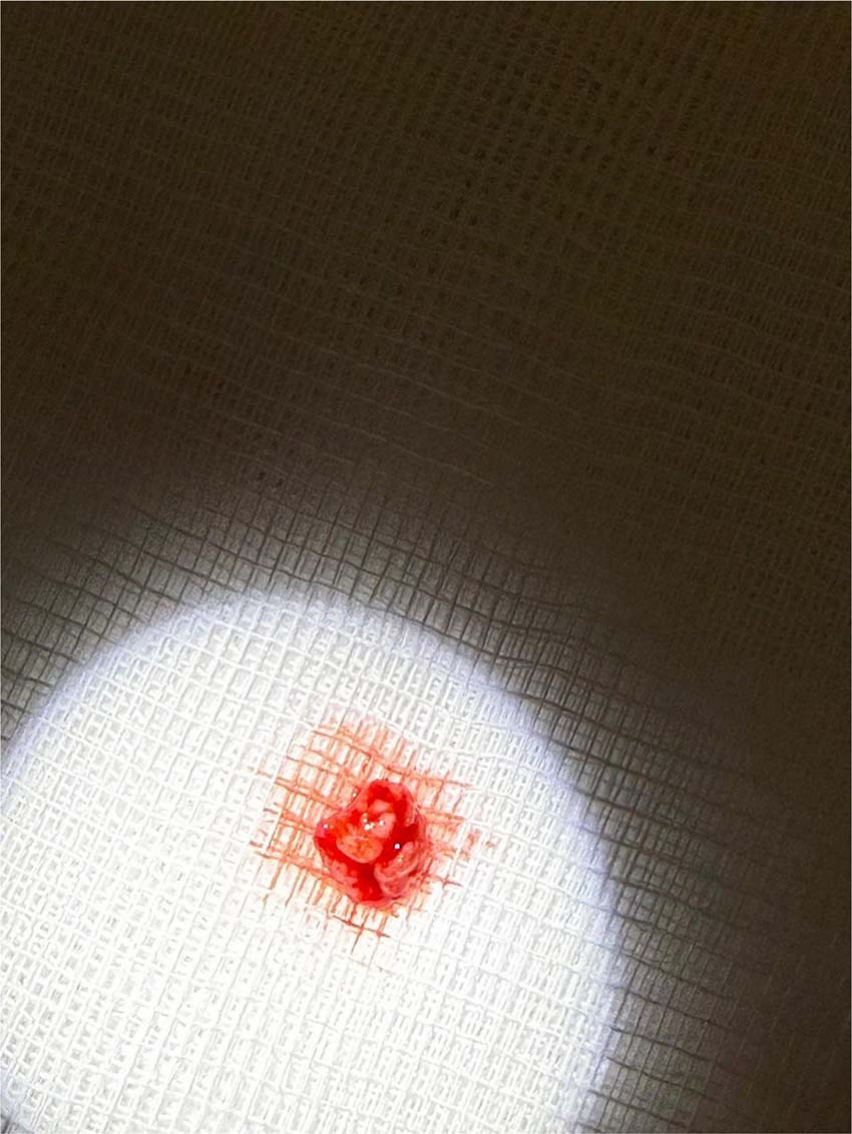
Excised specimen of the lesion placed on sterile gauze immediately after removal. The mass appears as a lobular red soft-tissue fragment.

**Figure 4:**
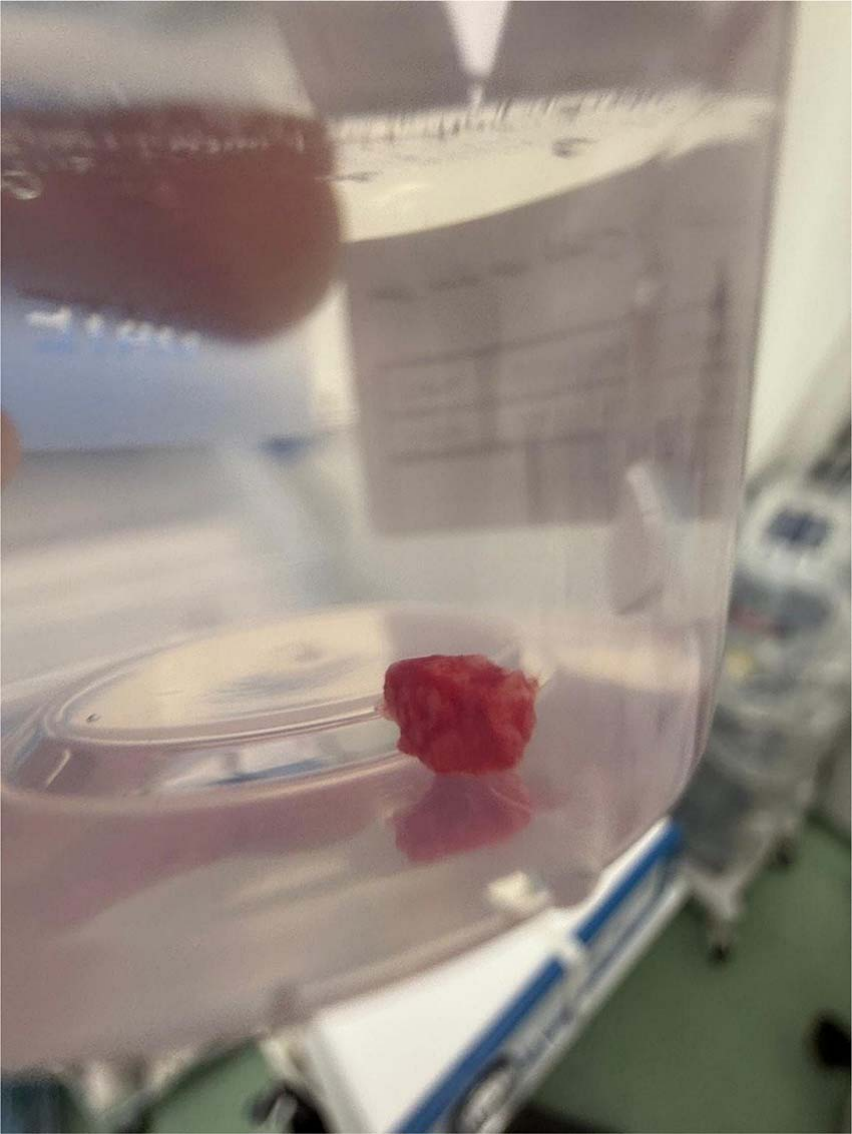
The excised lesion stored in a formalin container for pathological examination. The globular tissue specimen corresponds to the removed PG.

Initial management consisted of direct pressure and topical adrenaline to control bleeding, intravenous fluid resuscitation, and reversal of anticoagulation. Coagulation studies were consistent with apixaban use. Once partial hemostasis was achieved, the patient was taken to the operating theater. Under local anesthesia with conscious monitoring, the lesion was completely excised at its base on the soft palate using electrocautery, achieving satisfactory hemostasis. The excised specimen appeared as a lobulated red soft-tissue mass (Fig. 3) and was placed in formalin for histopathological assessment (Fig. 4).

Postoperatively, the patient’s vital signs stabilized and no further bleeding occurred. Apixaban was resumed 24 hours after the procedure without complications. The patient was discharged on postoperative Day 2. Histopathological examination demonstrated lobular aggregates of capillaries within a fibromyxoid stroma and overlying ulcerated epithelium, consistent with a lobular capillary hemangioma (PG). No dysplasia or malignancy was present. At two-month follow-up, the patient remained well with complete healing of the surgical site and no evidence of recurrence.

## Discussion

PG is a benign reactive lesion that represents an exaggerated healing response to various stimuli [3]. Common triggers include local trauma, chronic irritation, poor oral hygiene, and hormonal factors (pregnancy) [5]. The lesion typically presents as a rapidly growing red nodule that bleeds easily [1]. Gingival involvement is most frequent, reflecting plaque or calculus irritation [6]. Extra gingival occurrences are uncommon; only a few cases have reported PG on the palate [4]. Our case adds to this spectrum by documenting a large soft palate PG in an elderly patient.

In this patient, anticoagulation exacerbated hemorrhage. PGs have a known propensity to bleed due to their high vascularity [1]. In patients on Apixaban or other anticoagulants, minor trauma to a vascular lesion can lead to severe hemorrhage requiring urgent intervention. Management in such cases involves stabilizing the patient, controlling bleeding and definitive excision of the lesion. Surgical excision with cauterization at the base is the treatment of choice [5]. Complete removal of the sessile lesion, including its broad base of attachment, is crucial to minimize the risk of recurrence. No additional therapy was required in our patient, and there was no recurrence at follow-up, in line with the generally excellent prognosis of PG after adequate excision [2].

Histopathologically, PG shows lobular proliferation of capillaries set in a fibrous stroma, often with an overlying ulceration [1]. This finding differentiates it from other entities such as Kaposi’s sarcoma or angiosarcoma, which may present as vascular lesions in the oropharynx but have atypical cells and malignant features. Clinicians should include PG in the differential diagnosis of vascular soft palate lesions, particularly when evaluating rapidly growing or bleeding masses in anticoagulated patients. Accurate histopathological confirmation is essential, as the clinical appearance alone may mimic both benign and malignant processes [4].

This case highlights that PG, though benign, can present as an emergency in anticoagulated patients. Rapid recognition and surgical management are crucial. We recommend that in similar cases, Apixaban be held or reversed, and that excision be performed promptly under appropriate hemostatic measures. The case also underscores the importance of histological confirmation of diagnosis and follow-up for recurrence.
